# Supplementary material for: Biodiversity and Distribution of Reticulitermes in the Southeastern USA
Source: Insects. 2022 Jun 22;13(7):565. doi: 10.3390/insects13070565 (PMC9316241; doi:10.3390/insects13070565)
Supplement: Supplementary file 1 [file insects-13-00565-s001.zip › Figure S1.pdf]

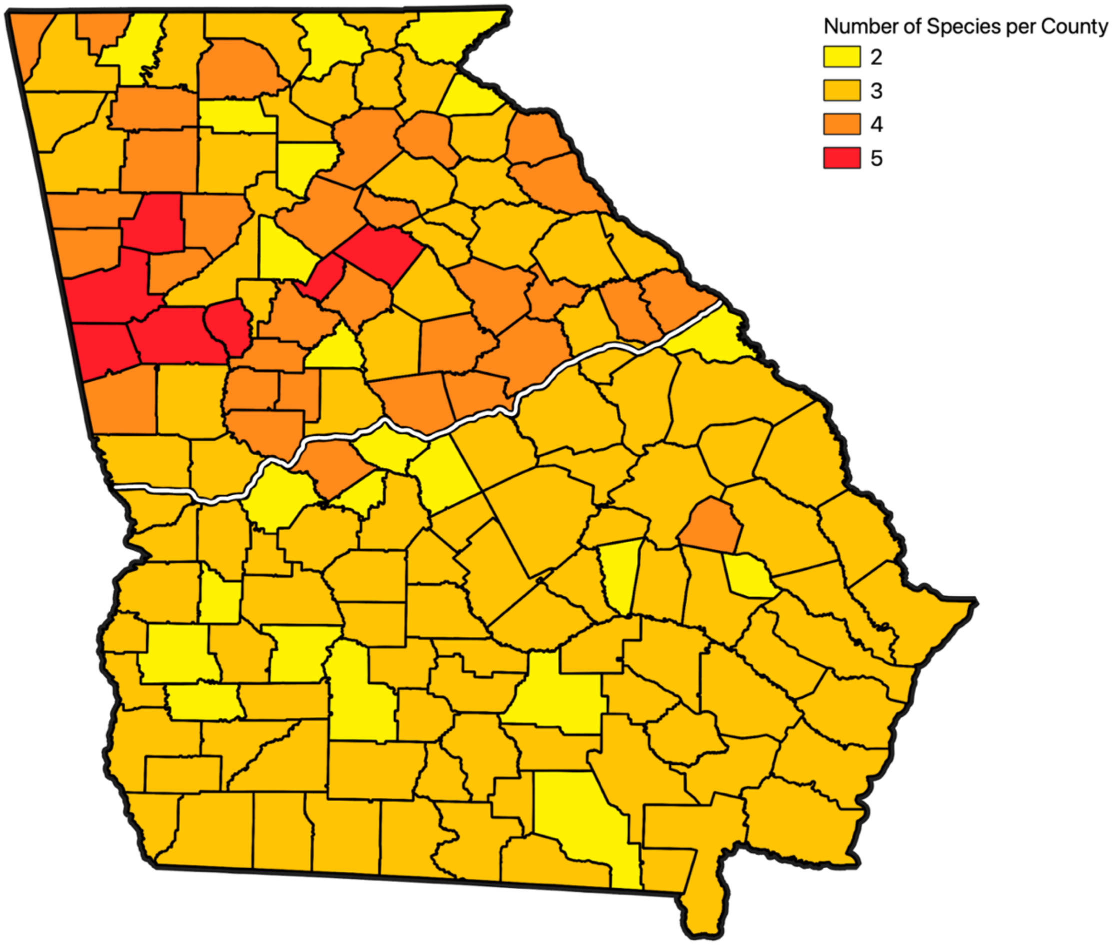

Figure S1. Map of the State of Georgia, USA by county showing the number of *Reticulitermes* species collected per county (n=159). The Fall Line is illustrated by a white line along the 32nd-33rd parallel.
