# Supplementary material for: Biodiversity and Distribution of Reticulitermes in the Southeastern USA
Source: Insects. 2022 Jun 22;13(7):565. doi: 10.3390/insects13070565 (PMC9316241; doi:10.3390/insects13070565)
Supplement: Supplementary file 1 [file insects-13-00565-s001.zip › Figure S2.pdf]

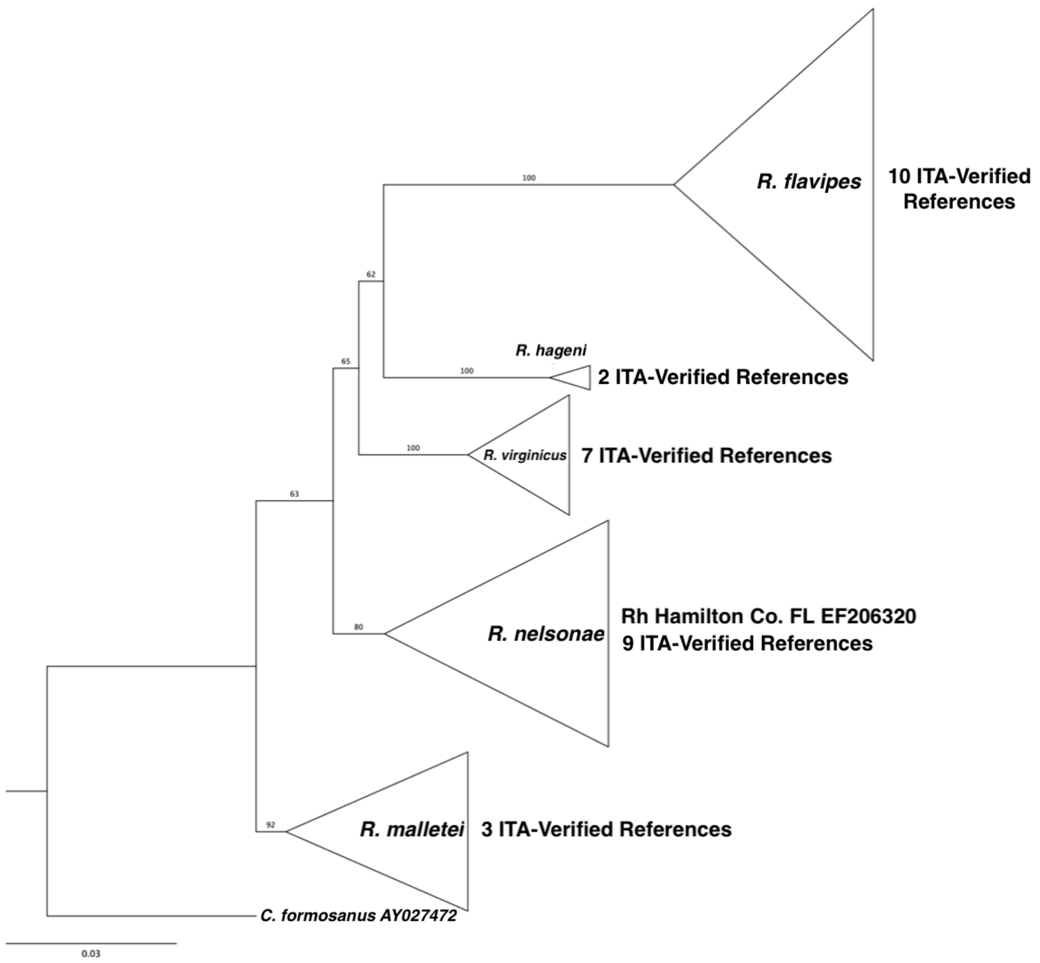

Figure S2. Results from 213 partial COI sequences including ITA-Verified reference sequence (TableS2) and the one anomalous GenBank accession attributed to Rh that grouped with Rn references in a collapsed Maximum-Likelihood GTR+G+I tree topology assembled using IQ-Tree v 2.6.12 [39] annotated in FigTree v. 1.4 (http://tree.bio.ed.ac.uk/software/figtree/). Branch numbers represent percentage of posterior probability from 1,000 UF Bootstrap iterations with support of ≥62% [57]. Full data available upon request from authors.
