# Supplementary material for: Biodiversity and Distribution of Reticulitermes in the Southeastern USA
Source: Insects. 2022 Jun 22;13(7):565. doi: 10.3390/insects13070565 (PMC9316241; doi:10.3390/insects13070565)
Supplement: Supplementary file 1 [file insects-13-00565-s001.zip › Supplemental Sample Preparation.pdf]

Sample Preparation

Samples were air-dried on filter paper (MilliporeSigma, Burlington, MA, USA) for 1 hr and added to 150 μL chilled (4°C) Nuclei Lysis (NL) solution (Promega, WI, USA) in a 1.7 mL microcentrifuge tube (Thermo Fisher Scientific, Waltham, MA). Samples were pulverized in NL solution with a sterilized pestle, combined with 17 μL of 20 mg/mL water suspended solution of Proteinase K (New England Biolabs), and incubated for 2 hr at 65°C in a dry bath incubator (Thermo Fisher Scientific, Waltham, MA). TE buffer suspended RNAse A solution (5 μL of 4 mg/mL) (Promega, WI) was added to the mixture and incubated for 30 mins at 37°C. Protein Precipitation solution (200 μL; Promega, WI, USA) was added to the mixture, chilled at 4°C for 5 mins, and centrifuged for 4 mins at a maximum speed of 12,400 RPM (Thermo Fisher Scientific, Waltham, MA). The supernatant (700 μL) was removed and added to a new 1.7 mL microcentrifuge tube. Molecular grade isopropanol (600 μL) (Thermo Fisher Scientific, Waltham, MA) was added to the 1.7 mL tube and centrifuged at maximum speed. The resulting supernatant was removed (1100 μL), and a second precipitation was performed with 600 μL of 70% ethanol (Decon Laboratories Inc, King of Prussia, PA) with a 20 min centrifuge cycle at maximum speed. The ethanol supernatant was removed, and the pellet was air-dried for 1 hr and resuspended in 40 μL of DNA rehydration solution (Promega, WI) for an hour at 65°C.
