# Supplementary material for: Biodiversity and Distribution of Reticulitermes in the Southeastern USA
Source: Insects. 2022 Jun 22;13(7):565. doi: 10.3390/insects13070565 (PMC9316241; doi:10.3390/insects13070565)
Supplement: Supplementary file 1 [file insects-13-00565-s001.zip › Table S1.pdf]

Table S1. List of publications that provide information on subterranean termite species distributions in the Eastern United States including the following 5 species; Rf= *R. flavipes*, Rv= *R. virginicus*, Rh= *R. hageni*, Rm= *R. malletei*, Rn= *R. nelsonae*, reported by state (standard abbreviations), sample collection method and corresponding citation. WL= samples from wildlands wood on ground or research plots; S = samples from pest management professionals; T= samples from light trap surveys; MC= samples from museum collections; Li= literature search; NM= Not mentioned.

**Regional Publications**			
**State**	***Reticulitermes spp* reported**	**Collection methods**	**Citation**
GA FL SC NC VA WV KY TN AL MS	Rf	WL; S	[54]
TX FL SC VA	Rh		
GA LA FL NC VA	Rv		
TX LA AK MS AL GA FL SC NC TN KY VA WV	Rf	Li	Light and Pickens, 1946
LA AK MS AL GA TN KY SC NC FL VA WV	Rh		
LA AK MS AL GA TN KY SC NC FL VA WV	Rv		
TX LA MS AK TN GA FL SC NC KY VA WV IL WI MI IN OH PA NY DW MD VT MA CT ME	Rf	NM	Snyder, 1946
LA MS GA FL SC NC VA WV KY MD DE IL IN OH	Rh		
LA MS GA FL SC NC VA WV KY MD DE IL IN OH	Rv		
TX LA MS AK TN GA FL SC NC KY VA WV IL WI MI IN OH PA NY DW MD VT MA CT MO	Rf	NM	[25]
LA AK MS AL GA FL SC NC TN KY VA WV	Rh		
LA AK MS AL GA FL SC NC TN KY VA WV	Rv		
FL LA TX	Rf	S	[22]
LA	Rh		
FL LA	Rv		
GA SC NC AL TN MS VA WV	Rf	WL	[58]
GA TN	Rv		
GA AL	Rm		
AL	Rn		
GA SC NC AL TN MS WV VA KY	Rf	WL	Hyseni and Garrick, 2019
GA KY VA AL SC NC	Rv		
AL SC	Rm		
**State Surveys**			
**State**	***Reticulitermes spp* reported**	**Collection methods**	**Citation**
Alabama	Rf, Rh, Rv, Rm	WL MC	[61]
Florida	Rf, Rh, Rv	S, Li	[24]

	Rf, Rh, Rv	S	Scheffrahn et al., 1988
	Rf, Rh, Rv	S	[53]
Georgia	Rf, Rv	N/A	(Light, 1946)
	Rf, Rv, Rh	WL MC	Scheffrahn et al., 2001
	Rf, Rv, Rh, Rm, Rn	WL	[27]
Louisiana	Rf, Rv, Rh	S	[63]
Mississippi	Rf, Rv	WL	Wang and Powell, 2001
South Carolina	Rf, Rh, Rv	WL S MC T	Hathorne et al., 2000
Texas*	Rf, Rv, Rh	S MC	Howell et al., 1987

* Rt= *R. tibialis* also reported in Howell et al., 1987.
