# Supplementary material for: Biodiversity and Distribution of Reticulitermes in the Southeastern USA
Source: Insects. 2022 Jun 22;13(7):565. doi: 10.3390/insects13070565 (PMC9316241; doi:10.3390/insects13070565)
Supplement: Supplementary file 1 [file insects-13-00565-s001.zip › Table S2.pdf]

Table S2. GenBank Accession numbers for the ITA-Verified COI and COII sequences termed Reference Sequence along with collection date and locale. The reference sequences were used to designate survey samples to a species designation using a GTR + G + I ML program.

**COI**			
**Species**	**Accession No.**	**Date Collected**	**Location**
C. formosanus	AY027472	2001	USA
R. flavipes	JN207470	2/6/07	Sapelo Island, GA, USA
	JN207471	2007	Sapelo Island, GA, USA
	JN207472	11/13/07	Sapelo Island, GA, USA
	JN207473	11/23/07	Sapelo Island, GA, USA
	JN207474	10/23/07	Athens, GA, USA
	JN207475	4/13/09	Athens, GA, USA
	JN207476	4/25/09	Athens, GA, USA
	JN207477	4/21/09	Athens, GA, USA
	F182	3/12/14	Athens, GA, USA
	F192	3/20/19	Athens, GA, USA
R. hageni	JN207483	2007	Athens, GA, USA
	H13	8/20/13	Clarke, Co. USA
R. virginicus	JN207478	11/13/07	Sapelo Island, GA, USA
	JN207479	1/30/08	Sapelo Island, GA, USA
	JN207480	2007	Athens, GA, USA
	JN207481	10/23/07	Athens, GA, USA
	JN207482	10/23/07	Athens, GA, USA
	V41	5/10/16	Athens, GA, USA
	V44	5/13/17	Athens, GA, USA
R. nelsonae	JN207486	2/6/07	Sapelo Island, GA, USA
	JN207487	2007	Sapelo Island, GA, USA
	JN207488	1/30/08	Sapelo Island, GA, USA
	JN207489	2/6/07	Sapelo Island, GA, USA
	JN207490	2/6/07	Sapelo Island, GA, USA
	JN207491	2/6/07	Sapelo Island, GA, USA
	N5	2/6/07	McIntosh Co, GA, USA
	N9	3/31/11	Branford, FL, USA
	N10	4/18/13	McIntosh, GA, USA
R. malletei	JN207484	2007	Athens, GA, USA

	JN207485	10/23/07	Athens, GA, USA
	M47	5/14/12	Athens, GA, USA

COII

Species	Accession #	Date Collected	Location
C. formosanus	AY683218	6/12/03	Savannah, GA, USA
R. flavipes	JF796216	2009	Athens, GA, USA
	JF796217	8/25/09	Thomasville, GA, USA
	JF796218	7/6/09	USA
	JF796219	3/31/10	Branford, FL, USA
	JF796220	11/5/10	Branford, FL, USA
	F2	4/19/96	Pike Co., GA, USA
	F24	3/28/02	Clarke Co., GA, USA
	F42	4/19/12	Clarke Co., GA, USA
	F50	2/13/13	Clarke Co., GA, USA
	F51	2/20/13	Clarke Co., GA, USA
	F52	2/28/13	Athens, GA, USA
	F58	4/13/13	Athens, GA, USA
	F174	3/10/13	Athens, GA, USA
	F182	3/12/14	Athens, GA, USA
	F192	3/20/19	Athens, GA, USA
	F193	4/27/19	Athens, GA, USA
	FJB	3/4/21	Athens, GA, USA
R. hageni	AF107486	8/97	Barnesville, GA, USA
	JF796224	2007	Athens, GA, USA
	JF796225	2007	Athens, GA, USA
	EU689026	2008	USA
	H11	8/5/07	Athens, GA, USA
	H13	8/20/13	Clarke Co., GA, USA
	H18	8/15/16	Athens, GA, USA
	H19	8/15/16	Athens, GA, USA
R. virginicus	JF796234	5/26/05	Madison, FL, USA
	JF796223	2009	Thomasville, GA, USA
	JF796222	7/5/09	Sapelo Island, GA, USA
	JF796221	5/30/08	Athens, GA, USA

	V41	5/10/16	Athens, GA, USA
	V44	5/13/17	Athens, GA, USA
	V46	5/14/17	Athens, GA, USA
	V47	5/14/17	Athens, GA, USA
R. nelsonae	JF796235	N/A	Sapelo Island, GA, USA
	JF796229	2009	Croatan NF, NC, USA
	JF796233	12/20/06	Apopka, FL, USA
	JF796232	6/18/09	GA, USA
	JF796231	3/31/10	Branford, FL, USA
	JF796230	2009	Thomasville, GA, USA
	EU689013	2008	GA, USA
	N5	2/6/07	McIntosh Co, GA, USA
	N9	3/31/11	Branford, FL, USA
	N10	4/18/13	McIntosh, GA, USA
	N11	4/8/13	McIntosh Co, GA, USA
	N12	5/29/10	Athens, GA, USA
R. malletei	JF796226	10/23/07	Athens, GA, USA
	JF796228	2009	NC, USA
	GU550074	N/A	USA
	JF796227	5/21/10	Athens, GA, USA
	M11	4/26/00	Winston, MS, USA
	M47	5/14/12	Athens, GA, USA
	M49	6/24/12	Athens, GA, USA
	M62	5/26/14	Athens, GA, USA
	M44	4/22/12	Athens, GA, USA
