# Supplementary material for: Biodiversity and Distribution of Reticulitermes in the Southeastern USA
Source: Insects. 2022 Jun 22;13(7):565. doi: 10.3390/insects13070565 (PMC9316241; doi:10.3390/insects13070565)
Supplement: Supplementary file 1 [file insects-13-00565-s001.zip › Table S3.pdf]

Table S3. Citations for the 17 *Reticulitermes* field surveys in the SE USA published between 2012-2022 including species mentioned, year published, and index to the citation.

Species Mentioned	Year	Citation
Rf, Rm	2012	Fredericks, 2012
Rf, Rv, Rh	2012	Blount, 2012
Genus only	2013	Little et al., 2013
Genus only	2014	Ulyshen, 2014
Genus only	2014	Little et al., 2014
Genus only	2014	Riggins et al., 2014
Rf, Rv, Rh, Rm, Rn	2015	Janowiecki, 2015
Rf, Rv, Rh, Rm	2015	Janowiecki and Szalanski, 2015
Rf	2016	Su et al., 2016
Genus only	2016	Clay et al., 2016
Rf, Rv, Rm	2019	Hyseni and Garrick, 2019
Rf	2019	Hyseni and Garrick, 2019
Rf, Rv, Rm	2020	Hyseni, 2020
Rf, Rv, Rh, Rm, Rn	2021	Janowiecki and Vargo, 2021
Rf, Rv, Rh	2021	[49]
Rf	2021	Aguero et al., 2021
Rf, Rv	2021	Shults et al., 2021
