# Supplementary material for: Biodiversity and Distribution of Reticulitermes in the Southeastern USA
Source: Insects. 2022 Jun 22;13(7):565. doi: 10.3390/insects13070565 (PMC9316241; doi:10.3390/insects13070565)
Supplement: Supplementary file 1 [file insects-13-00565-s001.zip › Table S4.pdf]

Table S4. COI, COII, and 16S accessions included in Maximum-Likelihood phylogenies with ITA-verified references that did not align in a clade matching the GenBank species designation. Table includes accession, collection location, GenBank species designation and updated based- on-grouping-with-ITA species designations, and publications referencing those aberrant accessions.

COI				
**Accession(s)**	**Collection Locale**	**GenBank Species Designation**	**ITA Species Designation**	**Publications**
EF206320*	Hamilton, Co. FL	*R. hageni*	*R. nelsonae*	[29, 70] Bourguignon et al., 2014
**COII**				
**Accession(s)**	**Collection Locale**	**GenBank Species Designation**	**ITA Species Designation**	**Publications**
EF206320*	Hamilton, Co. FL	*R. hageni*	*R. nelsonae*	[29, 70] Bourguignon et al., 2014; Wang et al., 2016; Lee et al., 2017; Li et al., 2018
AF525328*	Cumberland Island, GA & AR[t]	*R. hageni*	*R. nelsonae*	[30, 64] Austin et al., 2004; Nobre et al., 2006
AY808089*	Seminole Co. FL	*R. hageni*	*R. nelsonae*	[50]
AY808088*	Brevard, FL	*R. hageni*	*R. nelsonae*	[50]
AY536419	Jasper Pulaski IN	*R. virginicus*	*R. nelsonae*	Direct GenBank Submission
AY536418	Jasper Pulaski IN	*R. virginicus*	*R. nelsonae*	Direct GenBank Submission
AY536424	Indiana Dunes, Paradise Valley Ridge, IN	*R. virginicus*	*R. nelsonae*	Direct GenBank Submission
AY536422	Jasper Pulaski, IN	*R. virginicus*	*R. nelsonae*	Direct GenBank Submission
AY536423	Jasper Pulaski, IN	*R. virginicus*	*R. nelsonae*	Direct GenBank Submission
AY536425	Indiana Dunes, Paradise Valley Ridge, IN	*R. virginicus*	*R. nelsonae*	Direct GenBank Submission
AY536417	Jasper Pulaski, IN	*R. virginicus*	*R. nelsonae*	Direct GenBank Submission
AY536421	Jasper Pulaski, IN	*R. virginicus*	*R. malletei*	Direct GenBank Submission
**16S****				
**Accession(s)**	**Collection Locale**	**GenBank Species Designation**	**ITA Species Designation**	**Publications**
AY808122	Brevard, FL	*R. hageni*	*R. nelsonae*	[50]
AY808123	Seminole Co. FL	*R. hageni*	*R. nelsonae*	[50]
AY257230- AY257235	Brazos, TX Cumberland Island, GA	*R. hageni*	*R. nelsonae*	[64, 49] McKern et al., 2006

* Rh accessions identified to Rn mentioned in the published description of Rn in 2012 [27].

**All 16S accessions were only those we could cross list with published COII sequence because the aforementioned accessions could not be aligned with multiple sequences using Muscle *3.8.425* for phylogenetic analysis.

[t] This same haplotype was also attributed to samples collected in Arkansas (Austin et al., 2004).
