# Supplementary material for: Biodiversity and Distribution of Reticulitermes in the Southeastern USA
Source: Insects. 2022 Jun 22;13(7):565. doi: 10.3390/insects13070565 (PMC9316241; doi:10.3390/insects13070565)
Supplement: Supplementary file 1 [file insects-13-00565-s001.zip › Table S5.pdf]

Table S5. Accession numbers for the 16 COII sequences used to construct the global comparison phylogeny in Figure 5 along with collection date and locale. These sequences were used alongside ITA-Verified reference sequence (Table S2) to designate accessions to a species designation using a GTR + G + I ML phylogeny.

COII			
**Species**	**Accession No.**	**Submission Date**	**Location**
R. balkanensis	KM245783.1	7/14/14	Schinias, Greece
	KM245784.1	7/25/14	Nea Makri, Greece
R. urbis	DQ866972.1	7/26/06	Igoumenitsa, Greece
	JQ231196.1	12/3/11	Castellaneta, Italy
R. speratus	KM245821.1	7/25/14	Kamigamo, Kyoto, Japan
	KM245819.1	7/25/14	Motoyama, Kochi, Japan
R. chinensis	AB050705.1	11/1/2000	Beijing, China
	JX142148.1	6/6/12	Changping, Beijing, China
	FJ423454.1	10/28/08	Beijing, China
R. kanmonensis	KM245812.1	7/25/14	Ejio Park, Yamaguchi, Japan
	KM245811.1	7/25/14	Ejio Park, Yamaguchi, Japan
R. hesperus	DQ018960.1	4/26/05	CA, USA
	AY623447.1	5/12/04	CA, USA
	KM245769.1	7/25/14	Placerville, CA, USA
	KM245770.1	7/25/14	Novato, CA, USA
R. tibialis	AY808094.1	11/1/04	AZ, USA
	AF525355.1	6/28/02	AZ, USA
	HM208248.1	5/6/10	CA, USA
R. lucifugus	JQ231194.1	12/3/11	Pisticci, Marina, Italy
	JQ231192.1	12/3/11	Marina di Lesina, Italy
	EF591507.1	5/3/07	Corsica, Bastia, France
R. banyulensis	KM245779.1	7/25/14	Teruel, Spain
	KM245778.1	7/25/14	Cassis, France
R. grassei	MN107083.1	7/24/19	France
	MT188739.1	3/12/20	Foret Coubre, Point Espagnols, France
